# Tactile stimulation reduces fear in fish

**DOI:** 10.3389/fnbeh.2013.00167

**Published:** 2013-11-22

**Authors:** Annett Schirmer, Suresh Jesuthasan, Ajay S. Mathuru

**Affiliations:** ^1^Department of Psychology, National University of SingaporeSingapore, Singapore; ^2^Duke/NUS Graduate Medical SchoolSingapore, Singapore; ^3^LSI Neurobiology/Ageing Programme, National University of SingaporeSingapore, Singapore; ^4^Institute of Molecular and Cell BiologySingapore, Singapore; ^5^Department of Physiology, National University of SingaporeSingapore, Singapore

**Keywords:** zebrafish, schreckstoff, stress, cortisol, emotion, social

## Abstract

Being groomed or touched can counter stress and negative affect in mammals. In two experiments we explored whether a similar phenomenon exists in non-mammals like zebrafish. In Experiment 1, we exposed zebrafish to a natural stressor, a chemical alarm signal released by injured conspecifics. Before moving them into an observation tank, one group of fish was washed and then subjected to a water current that served as the tactile stimulus. The other group was simply washed. Fish with tactile treatment demonstrated fewer fear behaviors (e.g., bottom dwelling) and lower cortisol levels than fish without. In Experiment 2, we ascertained a role of somatosensation in these effects. Using a similar paradigm as in Experiment 1, we recorded fear behaviors of intact fish and fish with damaged lateral line hair cells. Relative to the former, the latter benefited less from the tactile stimulus during fear recovery. Together these findings show that tactile stimulation can calm fish and that tactile receptors, evolutionarily older than those present in mammals, contribute to this phenomenon.

## Introduction

Threats, even if transient, induce an unpleasant state. The narrow escape from a car accident, the passing of a shadowy figure in the dark, or the temporary fright of having lost something dear can chill us to the bone and may linger with us for hours or days. What speeds recovery is the presence of friends or loved ones. Moreover, being spoken to, but perhaps more importantly, being affectionately touched can go a long way in restoring one’s sense of calm. Here, we explored calming through touch in terms of its ubiquity in the animal kingdom and its biological underpinnings.

Human touch has a long history in healing and medical therapy. In ancient Greece, Hippocrates (ca 460–370 BC), the father of Western medicine, hailed “rubbing” as an important physician’s skill (Kline, 2004; Grammaticos and Diamantis, 2008). Modern empirical work corroborated this by showing that a few minutes of massage reduce stress hormones and increase the circulation of chemicals that counteract physiological arousal (Morhenn et al., 2012). Moreover, repeated massages over the course of days or weeks were found helpful in a number of conditions ranging from insomnia (Oliveira et al., 2012) to depression and cancer (Noto et al., 2010; Krohn et al., 2011).

Importantly, not only extended massages confer benefits. Simple touches, such as holding hands or tapping another’s forearm, can dispel threat and promote calm. Among others this was shown in an infant study using the still face procedure to induce stress (Feldman et al., 2010). In this study, mothers engaged with their infants and then suddenly remained still and non-responsive for 2 min. Infants confronted in this way showed signs of distress such as fussing and crying as well as increased physiological stress reactivity. Notably, these responses were dampened when their otherwise “frozen” mother touched them as compared to when she did not touch them (Feldman et al., 2010). In research with adults, holding the hand of one’s partner or the experimenter reduced subjective and neural fear responses to a cue signaling the probability of an electric shock (Coan et al., 2006). In line with this, brain activation patterns to negative images of a violent accident or fight were modulated by touch indicating that touch made it easier for individuals to engage with disconcerting content (Schirmer et al., 2011).

In an effort to understand why and how touch dispels threat and promotes calm, researchers have explored the effect of touch in non-human animals. Looking at non-human primates, they found human-like effects. For example, macaques, after grooming, showed reduced heart-rate (Boccia et al., 1989; Aureli et al., 1999), reduced cortisol levels (Gust et al., 1993), and fewer displacement behaviors such as yawning or shaking, which are considered signs of stress (Schino et al., 1988). Because similar observations were made in non-primate species such as cows (Raussih et al., 2003; Schmied et al., 2010) and rats (Uvnas-Moberg et al., 1996; Maruyama et al., 2012), the underlying mechanisms may be understood as fairly basic somatosensory processes that predate human evolution.

But how ubiquitous are these processes across the animal kingdom? Moreover, to what extent do they depend on mammalian characteristics such as “groomable” fur or sociability? A recent study in fish suggests some answers to these questions. Taking advantage of an evolved symbiosis between so-called “cleaner fish” and their surgeon fish clients, Soares and colleagues (Soares et al., 2011), studied the effect of grooming in a scaled saltwater vertebrate. In the wild, surgeon fish seek out cleaners for the removal of ectoparasites. Sometimes, however, these cleaners cheat and, instead of removing parasites, eat the clients’ mucus, which is costly to regrow. Cleaners appease the conflict arising from such cheating by moving across the client’s back and massaging it with their fins.

Soares and colleagues explored the effect of such massaging by exposing surgeon fish to an artifical cleaner model that was either stationary or moving back and forth. Surgeon fish spent less time interacting with the former as compared to the latter model, which arguably offered more tactile stimulation. Moreover, the fish’s interactions were longer after previous confinement stress as compared to when they were unstressed. While interactions irrespective of the cleaner model reduced the fish’s cortisol levels, for the moving cleaner condition there was an additional correlation between the duration of these interactions and cortisol decline. Thus, the authors concluded that the cleaners’ massages buffer against stress and, together with the removal of parasites, confer benefits to the surgeon fish that outweigh occasional mucus costs.

Inspired by this work, we set out to determine whether the touch benefits bestowed on surgeon fish are a singular example that depends on a symbiotic cleaning relationship or whether they may be encountered in fish more generally. After all, tactile impressions, albeit non-social, are part and parcel of the typical aquatic environment coming largely from water movements induced by currents or nearby fishes. Here we conducted two experiments to test whether such movements function like a cleaner’s massage in that they are calming and help recover from threat.

## Experiment 1

We used zebrafish (*Danio rerio*), a small tropical freshwater fish, as subjects in three experimental groups. One group was exposed to Schreckstoff (SS; von Frisch, 1938, 1941), an alarm substance released by injured fish that induces fear in companions. A second group was exposed to SS followed by a water current that served as the tactile stimulus. A last group was used as control.

We predicted that fish exposed to SS would show heightened cortisol levels as well as species-typical fear behaviors such as diving to the bottom of the tank, freezing, and darting (Mathuru et al., 2012). Moreover, we expected these behaviors to be increased in subjects exposed to SS relative to control subjects and relative to subjects exposed to both SS and the water current. Because several factors are known to contribute to recovery from fear in fish (von Frisch, 1938, 1941), we made no prediction as to whether the present tactile stimulation would enable full or only partial recovery.

### Methods

#### Subjects

Thirty-one adult zebrafish (3 to 5 months old) in AB background served as subjects. The term “AB background” was coined in the 1980ies and stems from two original fish-lines, then referred to as A and B, that were inter-crossed to derive the newer AB line that is commonly used in laboratories as wild type fish.^1^ Eleven subjects (4 female and 7 male) completed the Schreckstoff only condition (SS-only), 10 subjects (5 female and 5 male) the condition that combined SS exposure with tactile stimulation (SS-touch), and 10 subjects (6 female and 4 male) served as controls (No-SS).

#### Procedure

Subjects were tested individually, between 14:00 and 18:00 in the afternoon. For testing, a net was used to remove fish from their home tanks into a small portable tank. This tank was brought into a darkened test room, where fish were again netted and moved into a beaker that contained 50 ml of ordinary tank-water or the same amount of water plus 50 μl SS. SS was prepared from euthanized zebrafish by inducing 7 to 10 shallow lesions with a Sharpoint knife (22.5° stab). Fish were then immersed into 2 ml of 20 μM Tris-Cl (pH 8.0) for 1 to 2 min and rocked on a rocker. The 2 ml crude extract was then centrifuged at 13.2 k rpm, filtered, and heated overnight at 95°C.

After a two-minute exposure to tank-water or tank-water with SS, subjects were again netted and moved into a washing chamber with 400 ml of tank-water. This served to remove potential traces of SS. Subjects in the SS-only and No-SS conditions were then again netted and directly transferred into an observation tank (30 cm × 6 cm × 13 cm −L × W × H), which was filled with tank-water to a depth of 10 cm. Subjects in the SS-touch condition were netted and moved into a beaker with 50 ml tank-water that was covered by a lid and placed in a sink. Fresh tank water was delivered into the beaker via a tube at a rate of approximately 15 ml/s for 30 s. Overflowing water left the beaker via the beak. Following this tactile stimulation, fish were moved into the observation tank where they remained for 10 min.

All subjects were tested individually. During their time in the observation tank, a white light LED (i-bar LED lamp, Koncept) provided uniform illumination from above, such that the tank area was visible and the experimenter was obscured in the dark. The subjects’ behavior was recorded on a MacBook with an external i-sight or an Agent v5 HD web camera placed ~50 cm in front of the tank. At the end of the experiment, subject fish were netted, immediately euthanized in ice water, dried using Kim wipes, and frozen in dry ice. Samples were kept at −70°C until thawed for cortisol extraction.

#### Behavioral analysis

Videos were re-digitized at 20 frames/s and the subject fish position was tracked semi-automatically using the “track objects” algorithm in MetaMorph 6.3. In-house software was then used to derive the fish’s position and swimming speed in mm/s. Based on these two measures, we computed the following dependent variables—time spent in the bottom quarter area of the tank, pausing episodes, freezing episodes, and darting episodes. Pausing episodes were defined as 1 s of immobility (speed < 3.5 mm/s) akin to short freezing. Freezing episodes were defined as pausing for 5 s. Darting episodes were defined as erratic swimming episodes where the swim speed exceeded the normal swim speed by 10 SD. Normal swim speed was defined as the average swim speed of five fish that underwent the same treatment as fish in the No-SS condition, except that they were not alone in the observation tank. Their swim speed was recorded for 10 min each.

#### Cortisol extraction and assay

Fish were thawed from −70°C, weighed, and decapitated. The fish body without the head was dissected into five pieces and divided into five 2 ml Eppendorf tubes. Two-hundred μl of 1X Phosphate Buffered Saline (PBS; pH 7.2) at 4°C was added to each tube and their content homogenized using an Ultra-Turrax Disperser (T10 basic; IKA). Cortisol was extracted in 1400 μl of Ethyl Acetate (Sigma; De Marco et al., 2013) by vortexing the tubes for 30 s, followed by centrifugation at 7000 G-force for 15 min at room temperature. Organic layer (top) that contained cortisol was collected in fresh tubes and left in the fume hood overnight to allow ethyl acetate to evaporate. Cortisol was reconstituted in 1 ml of 1X PBS and stored at 4°C. Enzyme Linked Immuno-Sorbent Assay (ELISA) was performed using Cayman cortisol measurement kit (Item # 500360) following the manufacturer’s instructions within 2–3 days of extraction and plates were read on a Tecan Infinite^®^ at 420 nm.

### Results

#### Behavior

**Figure 1:**
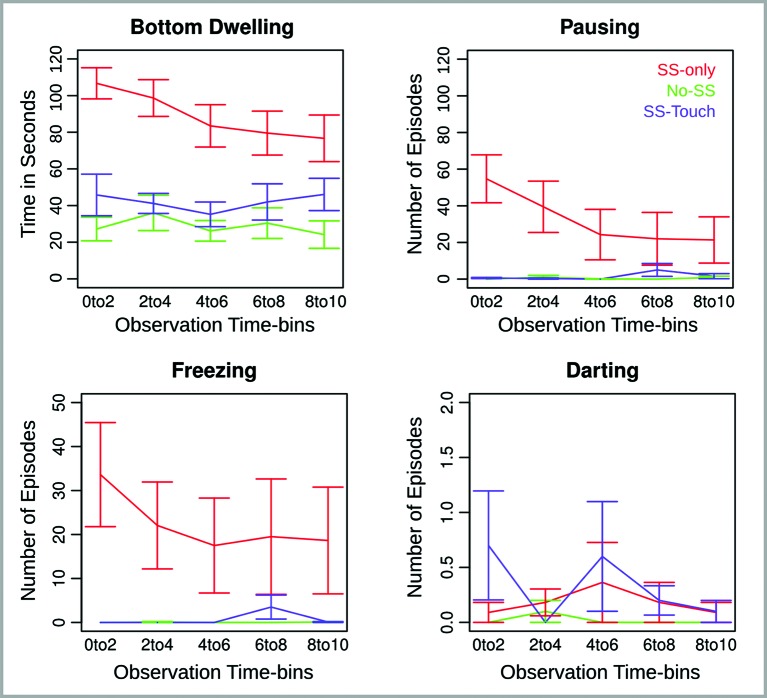
**Behavioral measures for Experiment 1 across five consecutive 2 min observation time bins.** Error bars reflect the standard error of the mean.

Behavioral data are illustrated in Figure 1. The four behavioral measures—bottom dwelling, pausing, freezing and darting—were subjected to separate ANOVAs with Observation Time-bin (0 to 2, 2 to 4, 4 to 6, 6 to 8, and 8 to 10 min) as a repeated measures factor and Group (SS-only, No-SS, SS-touch) as a between subjects factor. If follow-up tests of the factor Group concurred with our two predictions, they were conducted one-tailed. Otherwise, they were conducted two-tailed.

*Bottom Dwelling.* Analysis of bottom dwelling revealed an effect of Group (*F*(2,28) = 15.63, *p* < .0001), an effect of Observation Time-bin (*F*(4,112) = 3.55, *p* < .01), and an interaction of Group and Observation Time-bin (*F*(8,112) = 2.15, *p* < .05). Follow-up analyses for each time-bin indicated that the Group effect was largest at the beginning of the observation period and declined toward the end of it (*F*(2,28) = 22.08, 16.29, 13.09, 6.35, 6.89, *p*s < .01). Nevertheless, the Group effect remained significant and was further explored using the non-paired Welch *t*-test. Without exception, bottom dwelling was significantly more prominent in the SS-only condition relative to both the No-SS condition (*t*(18.1, 18.9, 14.4, 17.5, 16) = 7.5, 4.5, 4.5, 3.4, 3.5, *p*s < .01, one-tailed) and the SS-touch condition (*t*(17.1, 15.3, 15.9, 18.6, 17.4) = 4.3, 5, 3.6, 2.4, 1.9, *p*s < .05, one-tailed). Moreover, the latter two conditions differed only in the last time-bin where the SS-touch condition tended to elicit more bottom dwelling than the No-SS condition (*t*(17.5) = 1.9, *p* = .07, all other *p*s > .1, two-tailed).

*Pausing and Freezing.* Analysis of pausing revealed an effect of Group (*F*(2,28) = 6.2, *p* < .01), Observation Time-bin (*F*(4,112) = 3.4, *p* < .05), and a Group by Observation Time-bin interaction (*F*(8,112) = 3.8, *p* < .001). Follow-up analyses for each time-bin indicated that the Group effect was significant for the first and second time-bin (*F*(2,28) = 15.6, 6.9, *p*s < .01), marginally significant for the third time-bin (*F*(2,28) = 2.8, *p* = .08), and non-significant for the last two time-bins (*p*s > .1). In the first two time-bins, there was more pausing in the SS-only as compared to both the No-SS (*t*(10, 10.1) = 4.2, 2.7, *p*s < .05, one-tailed) and the SS-touch conditions (*t*(10, 10) = 4.1, 2.8, *p*s < .05, one-tailed). The latter two conditions were marginally different in the first (*t*(9) = 1.9, *p* = .08, two-tailed) and statistically comparable in the second time-bin (*p* > .1, two-tailed).

Analysis of freezing revealed only an effect of Group (*F*(2,28) = 5.9, *p* < .01). Fish in the SS-only condition froze longer than fish in the No-SS condition (*t*(54) = 4.4, *p*s < .0001, one-tailed). Importantly, a similar effect emerged when comparing fish in the SS-only condition to fish in the SS-touch condition (*t*(55.3) = 4.2, *p* < .0001, one-tailed). Fish in the SS-touch condition were not significantly different from fish in the No-SS condition (*p*s > .1, two-tailed).

*Darting.* Effects in the analysis of darting were non-significant (*p*s > .1).

#### Cortisol

Due to an oversight, four subjects of the No-SS condition were not stored at −70°C in due time, allowing us to analyze cortisol for only six subjects in this condition. Cortisol could be analyzed for all fish in the other two conditions.

**Figure 2:**
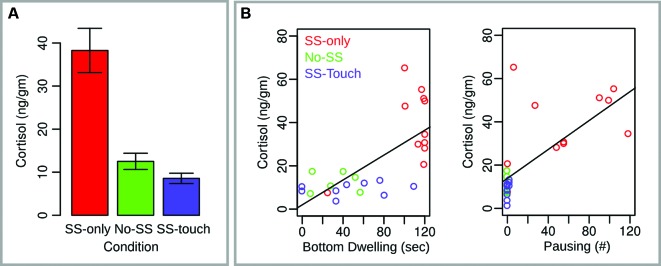
**Cortisol measures for Experiment 1.** **(A)** Mean cortisol values across conditions. Error bars reflect the standard error of the mean. **(B)** Scatter plots with regression lines indicating the relationship between cortisol and two fear behaviors, bottom dwelling and pausing.

Cortisol data are illustrated in Figure 2. An ANOVA with Group (SS-only, SS-touch, No-SS) as a between subjects factor was significant (*F*(2, 24) = 20.06, *p* < .0001). Follow-up unpaired Welch *t*-tests indicated that subjects in the SS-only condition had significantly higher cortisol levels than subjects in both the No-SS (*t*(12.4) = 4.7, *p* < .001, one-tailed) condition and the SS-touch condition (*t*(11.1) = 5.6, *p* < .0001, one-tailed). The latter two conditions did not differ (*p* > .1, two-tailed).

A general linear regression model with cortisol as the dependent variable and all four behavioral measures from the first time-bin as the independent variables was significant (*F*(4,22) = 9.3, *p* < .001, adjusted *R*^2^ = 0.56). Moreover, this effect was driven by a positive relationship between cortisol on the one hand and bottom dwelling (*t* = 2.24, *p* < .05) and pausing (*t* = 2.03, *p* = .05) on the other hand. Contributions of the remaining two independent variables were non-significant (*p* > .1).

### Discussion

Experiment 1 sought to explore whether non-social tactile stimulation arising from a water current can reduce fear in fish. We found fewer behavioral signs of fear in fish provoked with SS and subjected to a water current as compared to fish provoked with SS and subjected to still water only. Compared to the latter, the former fish spent less time in the bottom quarter of the tank and showed fewer arrestments in their movement as quantified by pausing and freezing episodes. Additionally, their cortisol levels were lower indicating that their body physiology more quickly recovered from threat.

Notably, differences between control fish and fish treated with SS plus water current were largely non-significant. Only a few tendencies emerged for bottom-dwelling and pausing that were limited to only a few observation minutes. Thus, it seems that the present tactile stimulation of 30 s was very effective in reducing fear suggesting that fish can gain benefits from the non-social tactile experiences that are part of their aquatic environment.

However, before attributing the present results to mechanosensation, one may wish to ascertain that they were not due to extraneous factors that varied between the experimental conditions. Exposing fish to water current affects not only their somatosensation. It also creates visual, auditory, and olfactory impressions that are absent in still water. To eliminate a potential role of these latter factors, we conducted a second experiment in which we impaired somatosensation in some fish and compared their fear recovery with that of intact fish.

## Experiment 2

Exposure to water current impacts all of a fish’s senses including its tactile sense. Research into how the zebrafish achieves the latter sense has highlighted three mechanisms. The first mechanism relies on trigeminal neurons innervating the head and thus relaying tactile stimulation of the head to the brain (Belousova et al., 1983). The second mechanism relies on Rohon-Beard neurons that innervate the rest of the body. Rohon-Beard neurons, however, are only a temporary receptor that gives way to dorsal root ganglia during the maturation from larvae to adult fish (Reyes et al., 2004). Together with the trigeminal neurons, Rohon-Beard neurons and their replacing ganglia are considered the primary mechanisms for tactile perception in zebrafish (Sagasti et al., 2005; Palanca and Sagasti, 2013).

Adding to these mechanisms, are hair cells in neuromasts arranged along the fish’s lateral line. Superficial neuromasts are situated on the body surface, whereas subdermal neuromasts are located along a channel system below the fish’s scales. Water flowing along the fish’s body surface and along the subdermal neuromast system can displace hair cells thereby giving rise to a third tactile mechanism. However, unlike the first two, this latter mechanism is more specific to sensing water current and to rheotaxis, a behavioral phenomenon whereby fish and other aquatic species orient towards a current enabling them to remain stationary without being swept away (Froehlicher et al., 2009; Suli et al., 2012).

With Experiment 2, we sought to explore the contribution of neuromast hair cells to the calming effect of water current observed in Experiment 1. To this end, we used two conditions already present in Experiment 1 that should replicate our earlier findings. Specifically, one group of fish was treated with SS-only, whereas a second group was treated with SS followed by 30 s of water current (SS-touch). We added a third group of fish (SS/Neo-touch) that, prior to the experiment, was exposed to Neomycin, an aminoglycoside antibiotic, causing temporary damage of neuromast hair cells and impairing the fish’s reliance on tactile information for rheotaxis (Suli et al., 2012).

Based on previous work and our own findings, we made the following two predictions. First, we predicted that fish in the SS-only condition would display more fear behaviors than fish in the SS-touch condition. Second, we predicted that fish in the SS/Neo-touch condition would display more fear behaviors than fish in the SS-touch condition. We made no concrete predictions regarding potential similarities between the SS/Neo-touch and the SS-only conditions. Although it was possible that Neomycin would abolish the tactile stimulation effect, we thought it prudent to assume this effect only reduced. One reason for this was that Neomycin treatment would only affect one tactile mechanism in the fish leaving trigeminal and dorsal root ganglia receptors intact. A second reason was that existing research is equivocal as to whether Neomycin damage is exhaustive and whether it affects primarily superficial or subdermal neuromasts (Song et al., 1995; Suli et al., 2012).

### Methods

#### Subjects

Thirty adult zebrafish (3 to 5 months old) in AB background served as subjects. Ten subjects (5 female and 5 male) were used for the SS-only condition, 10 subjects (5 female and 5 male) were used for the condition that combined SS exposure with tactile stimulation (SS-touch), and 10 subjects (7 female and 3 male) were used for the Neomycin treatment condition that again combined SS exposure with tactile stimulation (SS/Neo-touch).

#### Procedure

As regards the SS-only condition and the SS-touch condition, the procedure was identical to what was described for Experiment 1. The SS/Neo-touch condition was largely comparable to the SS-touch condition with the exception that fish were treated with Neomycin. To this end, they were removed from their home tanks within 24 h before the experiment and placed into a beaker with 200 ml tank-water and 100 μM of aminoglycoside (Neomycin sulfate, Calbiochem). The beaker was left in a dark room for 3 h. Subsequently, the fish were netted and moved into a container with tank-water only to wash off traces of the aminoglycoside. Finally, they were returned to their home tank. A test with 10 fish not included in the present study ensured that Neomycin treatment did not make fish fearful. Specifically, the Neomycin treated fish did not differ from untreated fish (i.e., No-SS group of Experiment 1) with respect to critical fear behaviors such as bottom-dwelling, freezing, pausing and darting (all *p*s > .1).

**Figure 3:**
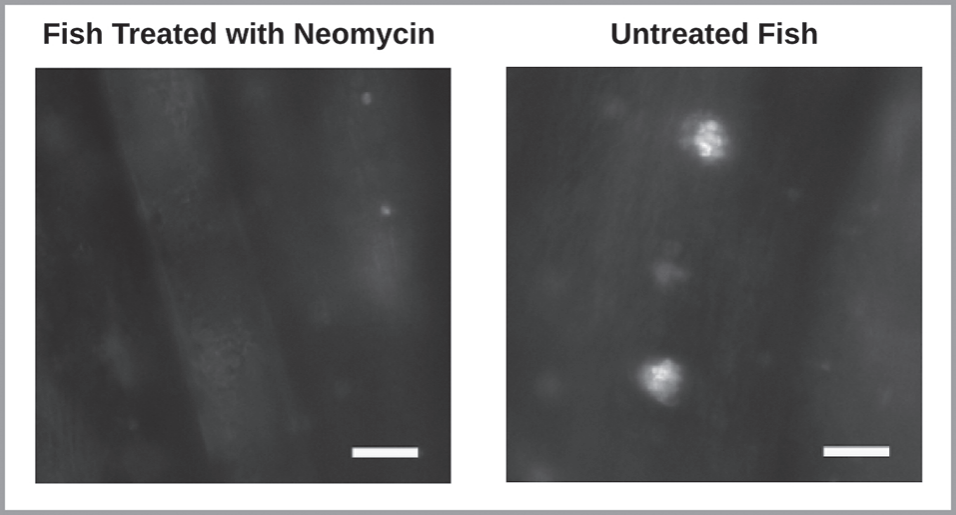
**Wide-field fluorescence images of two zebrafish tail fins.** Stained neuromast hair cells are absent in the Neomycin treated fish (left) and present in the untreated fish (right). Scale bar represents 75 μm.

To ascertain damage to the lateral line hair cells, a few Neomycin treated and untreated fish, not included in the behavior experiment, underwent 15 min exposure to 0.1 μM of the fluorescent dye 2-[4-(di- methylamino)styryl]-N-ethylpyridinium iodide (DASPEI; Molecular Probes, Eugene, OR), a vital dye that specifically stains hair cells within neuromasts. Fish were then anesthetized in MS222 (10 lg/ml, 3-aminobenzoic acid ethyl ester, methanesulfonate salt; Sigma, St. Louis, MO) and washed in tank-water after which their lateral line hair cells on the tail fin were observed under a fluorescence microscope with a 40X objective (Zeiss Examiner). As expected, this revealed damage to the superficial neuromast hair cells in Neomycin treated fish. In these fish, traces of the dye being taken up by the hair cells were barely visible. In contrast, dye uptake was clearly evident in untreated fish (Figure 3). Note that this technique did not allow us to test for potential damage to subdermal hair cells.

No cortisol measures were taken in Experiment 2.

### Results

#### Behavior

**Figure 4:**
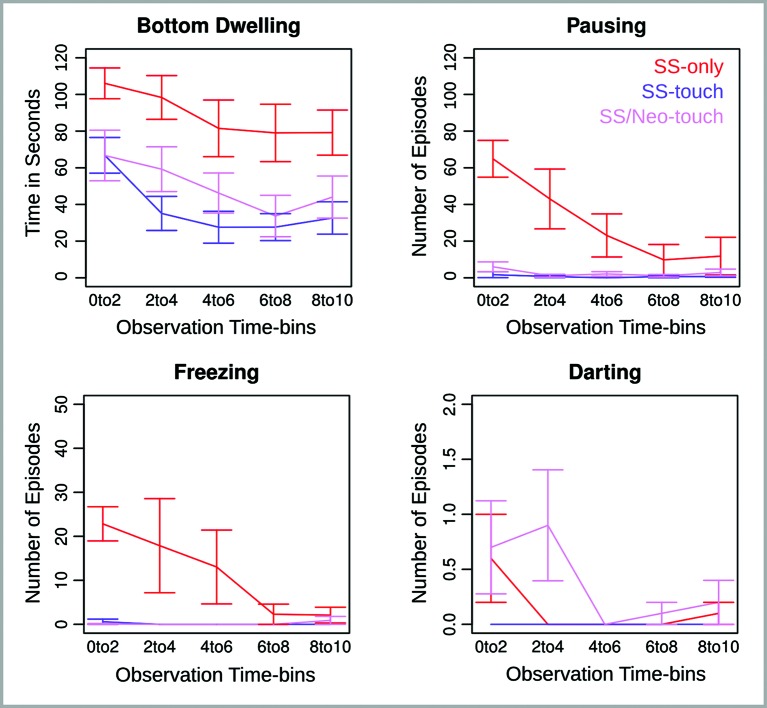
**Behavioral measures for Experiment 2 across five consecutive 2 min observation time bins.** Error bars reflect the standard error of the mean.

Behavioral data are illustrated in Figure 4. Again, the four dependent measures—bottom dwelling, pausing, freezing and darting—were subjected to separate ANOVAs with Observation Time-bin (0 to 2, 2 to 4, 4 to 6, 6 to 8, and 8 to 10 min) as a repeated measures factor and Group (SS-only, SS-touch, SS/Neo-touch) as a between subjects factor. If follow-up tests of the factor Group concurred with our two predictions, they were conducted one-tailed. Otherwise, they were conducted two-tailed.

*Bottom Dwelling.* Analysis of bottom dwelling revealed effects of Group (*F*(2,27) = 7.55, *p* < .01) and Observation Time-bin (*F*(4,108) = 11.7, *p* < .0001). The Group effect, as explored using an unpaired Welch *t*-test, indicated that in replication of Experiment 1 fish in the SS-only condition displayed more bottom dwelling than fish in the SS-touch condition (*t*(90.5) = 7.0, *p* < .0001, one-tailed). Additionally, the SS-only fish displayed more bottom dwelling than the SS/Neo-touch fish (*t*(97.5) = 4.9, *p* < .0001, two-tailed). Most importantly, however, the latter fish showed significantly more bottom dwelling than the SS-touch fish (*t*(93.5) = 1.7, *p* < .05, one-tailed) indicating that the somatosensory impairment reduced the calming effect of water current.

The Observation Time-bin effect indicated that bottom dwelling was maximal at the beginning of the observation period and declined towards the end (*t*(29) = 4.3, *p* < .001, two-tailed) pointing to a dissipation of fear over time that was evident across conditions.

*Pausing and Freezing.* Analysis of pausing and freezing revealed a Group effect (*F*(2,27) = 9.8, 8.2, *p*s < .01), an Observation Time-bin effect (*F*(4,108) = 9.3, 2.6, *p*s < .05), and a Group by Observation Time-bin interaction (*F*(8,108) = 7.4, 2.7, *p* < .05). When pursuing the interaction for pausing, we found that the Group effect was significant in the first and second time-bin (*F*(2,27) = 6.6, 3.5, *p*s < .05) but not later (*p*s > .1). In both the first and second time-bin, the SS-only condition produced significantly more pausing than the SS-touch condition (*t*(9.4, 9.0) = 6.2, 2.3, *p*s < .05, one-tailed) and the SS/Neo-touch condition (*t*(10.3, 9.0) = 5.7, 2.6, *p*s < .05, two-tailed). The critical comparison between the latter two conditions approached significance in the first time-bin (*t*(14.7) = 1.4, *p* = .09, one-tailed), but was non-significant in the second time-bin (*p* > .1).

By pursuing the interaction for freezing, we found that the Group effect was significant in the first time-bin (*F*(2,27) = 32.7, *p* < .0001), marginally significant in the second time-bin (*F*(2,27) = 2.8, *p* = .08), and non-significant in the remaining time-bins (*p*s > .1). In the first time-bin, freezing was greater in the SS-only condition as compared to both the SS-touch (*t*(9.4) = 5.6, *p* < .001, one-tailed) and the SS/Neo-touch conditions (*t*(9.0) = 5.8, *p* < .001, two-tailed). Freezing in the latter two conditions was comparable (*p* > .1).

*Darting.* Analysis of darting revealed a marginal Group effect (*F*(2,27) = 2.7, *p* = .08) and a significant Observation Time-bin effect (*F*(4,108) = 2.8, *p*s < .05). The interaction of both factors was non-significant (*p* > .1). Based on our hypotheses, we further explored the marginal Group effect and found that fish darted more in the SS-only as compared to the SS-touch condition (*t*(49) = 1.6, *p* = .05, one-tailed). Darting was comparable for the SS-only and the SS/Neo-touch condition (*p* > .1, two-tailed). Most importantly, darting was greater for the SS/Neo-touch condition as compared to the SS-touch condition (*t*(49) = 2.7, *p* < .01, one-tailed).

Follow-up analysis of the Observation Time-bin effect was non-significant (all *ps* > .1).

### Discussion

In Experiment 2, we set out to replicate the results of Experiment 1 and to ascertain a contribution of tactile mechanisms in the calming effect of water current. Both of these goals were achieved. As in Experiment 1, we observed large differences between fish exposed to SS as a function of whether or not these fish were subsequently treated with water current. Again, water current seemed to speed fear recovery. Adding to Experiment 1, we found that damage to neuromast hair cells impaired this effect. This was clearly evident for bottom-dwelling but also emerged tentatively for pausing and darting. For each of these measures, Neomycin-treated fish displayed or tended to display higher values than untreated fish exposed to water current. Thus, we conclude that tactile stimulation by water current reduces fear in fish and that lateral line receptors, together with other mechanisms not examined here, contribute to this effect.

## General discussion

Past research suggests that the psychological benefits arising from touch rely on basic mechanisms that are fairly independent of how touch is interpreted—mechanisms that humans share with other mammals. Here we asked whether these mechanisms are strictly mammalian in that they depend on mammalian fur and sociability.

### The role of fur

One recent study in surgeon fish implied that fur is unnecessary for touch to be beneficial (Soares et al., 2011). It showed that, following confinement stress, surgeon fish sought out interactions with a cleaner model and that these interactions aided in stress recovery. However, two aspects of this study make a role of tactile processing uncertain. For one, the interactions between surgeon fish and cleaners were not clearly described as tactile or physical in their relation to stress recovery. Second, and perhaps more importantly, the simple presence of a fish model, especially one that is moving, would have contributed to the observed effects. It is long established that animals living in shoals, herds, or groups gather when threatened to reduce danger to themselves and that such gathering in turn reduces fear (Hamilton, 1971). Among others, this was shown in fish by von Frisch in the last century (von Frisch, 1938). He found that frightened fish, when joining a calm shoal, became calm themselves. More recent work from our lab corroborated these findings and showed that companions are calming as long as they swim in a calm fashion themselves (Mathuru et al., unpublished). Importantly, the calming effect persists when companions are merely visible through glass and cannot be felt.

By directly applying a tactile stimulation to fish in the absence of companions, the present study addressed these concerns. Moreover, by demonstrating a specific contribution of somatosensation to the observed effects, it revealed original evidence that non-furry animals like fish experience psychological benefits from touch that compare to the touch effects observed in mammals and that suggest their origin predates mammalian evolution.

### The role of sociability

The present study also informs about a potential role of mammalian-like sociability in the psychological benefits of “being touched”. Specifically, two points of the present work suggest that such sociability may be unnecessary. First, the tactile stimulus employed here was non-social in the sense that it was not caused by other fish. Yet, the stimulus significantly calmed fish. Second, researchers typically exclude fish from their definition of “social species” (de Waal, 2011), which they consider endowed with a capacity for bonding with group members, mating partners, and/or offspring (but see Hinz et al., 2013; Mathuru et al., unpublished). Thus, while fish may live in shoals that on the surface compare to the groups in which many mammals like cows and humans live, they are believed to lack mammalian-like affectionate ties and associated social emotions (e.g., grief; de Waal, 2011). If one accepts this, then this together with the non-social stimulus used here indicate that the tactile facilitation of recovery from fear may be a largely non-social phenomenon.

Identifying touch effects as a non-social phenomenon may be surprising and potentially worrying. For one, the extant mammalian literature stresses the importance of touch for offspring care and social bonding. Touch in the form of grooming has been shown to be necessary for healthy offspring development and to influence physical growth and mental functioning (e.g., stress reactivity; Liu et al., 1997; Gonzalez et al., 2001; Cameron et al., 2005). Additionally, it has been established as a means to develop and maintain supportive relationships between group members (Henzi and Barrett, 1999). Through grooming, individuals increase the probability that their grooming partners become allies in later competition and conflict.

Further corroborating these findings is the discovery of a tactile receptor with properties that imply a specific social role (Olausson et al., 2002; Vrontou et al., 2013). This receptor, called a C-tactile afferent, is found only in the hairy skin of mammals. It seems optimized for the kind of touch and grooming mammals engage in as it responds to light pressure and stroking with maximal response rates at slow stroking velocities of ~4 cm per seconds. Interestingly, response rate of this receptor correlates positively with the pleasure that individuals experience from touch (Olausson et al., 2002; Morrison et al., 2011)—a property not found for other skin receptors and pointing to a key role of C-tactile afferents in the psychological benefits of touch (for a review see (Schirmer et al., under review).

How can the present results be reconciled with this? First, one may consider a few findings from the extant mammalian literature in which tactile benefits emerged from non-social touch. In rodents, for example, researchers found that manually brushing pups had comparable effects as maternal licking and grooming indicating that it was the tactile stimulation rather than the presence of their mother that was important (Gonzalez et al., 2001; Jutapakdeegul et al., 2003). In primates, Harry Harlow found that infant rhesus macaques deprived of their mother sought tactile comfort from a clothed wire frame and that such comfort dampened the adversities of social isolation (Harlow, 1958). In humans, one study directly compared touch from a friend with touch from a mechanical device and demonstrated a comparable influence on emotional responding (Schirmer et al., 2011). Thus, like in fish, tactile benefits in mammals do not necessitate a social relationship or agent.

Second, to reconcile our results with the extant literature one may consider potential benefits of non-social touch that may have contributed to such touch becoming a pleasurable experience. In fish, such benefits are easy enough to see. For one, tactile impressions from moving water may be pleasurable because they signal the presence of other fish and thus the safety of a shoal. Additionally, they may be pleasurable because moving water contains more oxygen than still water and may carry fresh food particles that the current detached from plants or rocks. In the case of zebrafish, these possibilities combine with the fact that they originate from streams in northeast Inda (Engeszer et al., 2007) suggesting that they find moving water particularly conducive to their way of life. Together these and other reasons may make water currents a positive stimulus that is effective in reducing fear.

When considering mammals, the benefits of non-social touch are not so obvious. Although humans and possibly other species derive pleasure from wind or other materials gently brushing against their skin, such brushing is not immediately useful. Unlike for aquatic animals, for terrestrial animals it is decoupled from oxygen or food and provides no information about the presence of companions. Why then should it be pleasurable? One possibility is that such brushing incidentally activates a more recent touch system that evolved specifically for the appreciation of social touch in the context of mammalian social bonding. Alternatively, however, it may be an aquatic heritage that is based on an evolutionary older and more general touch system co-opted by mammals for social touch. Although both possibilities are viable, we favor the latter one because it assumes evolutionary continuity and shows how an aquatic mechanism could have been shaped by the environmental conditions that animals met when transitioning from water to land.

## Conclusion

In sum, the present study showed that water current, a non-social tactile stimulus, alleviates fear in fish. This was evident from reduced fear behaviors such as bottom dwelling and a dampened physiological stress response as indicated by cortisol levels. Because a temporary dysfunction of neuromast hair cells impaired the water current effect, one can infer that it was at least partially mediated by the activation of lateral line hair cells. Together, these results provide first compelling evidence that fish derive benefits from tactile stimulation and suggest that parallel benefits observed in mammals may have aquatic roots.

## Conflict of interest statement

The authors declare that the research was conducted in the absence of any commercial or financial relationships that could be construed as a potential conflict of interest.

## Footnotes

^1^http://zfin.org/ZDB-GENO-960809-7
